# Lessons and Guidance from the Special Issue on Electronic Cigarette Use and Public Health

**DOI:** 10.3390/ijerph15071338

**Published:** 2018-06-26

**Authors:** Walton Sumner, Konstantinos Farsalinos

**Affiliations:** 1 Retired from Department of Internal Medicine, School of Medicine, Washington University, School of Medicine, Washington University, 660 S Euclid Ave, St. Louis, MO 63110, USA; wsumner@wustl.edu; 2 Department of Cardiology, Onassis Cardiac Surgery Center, Sygrou 356, 17674 Kallithea, Greece

## 1. Introduction

Electronic cigarette (e-cig) markets continue to grow, generating controversy regarding appropriate clinical and regulatory responses. On the one hand, the products have a disquieting gross similarity to the competing product, conventional cigarettes, must expose pulmonary tissue to many chemicals that are rarely breathed otherwise, and could expose young users to their first nicotine. On the other hand, cigarette smoking is one of the deadliest scourges in history, and it is vanishingly unlikely that e-cigs are equally risky. Our call for papers for this special issue listed diverse current topics related to e-cig chemistry and biology, which we need to understand to guide clinical use and regulation.

Readers should understand that we accept harm reduction as a valid tobacco control policy [1,2], and we expect e-cigs to have a role in smoking harm reduction. This is admittedly a minority position in many countries. Here are two arguments that compel us to endorse harm reduction.

**Figure 1:**
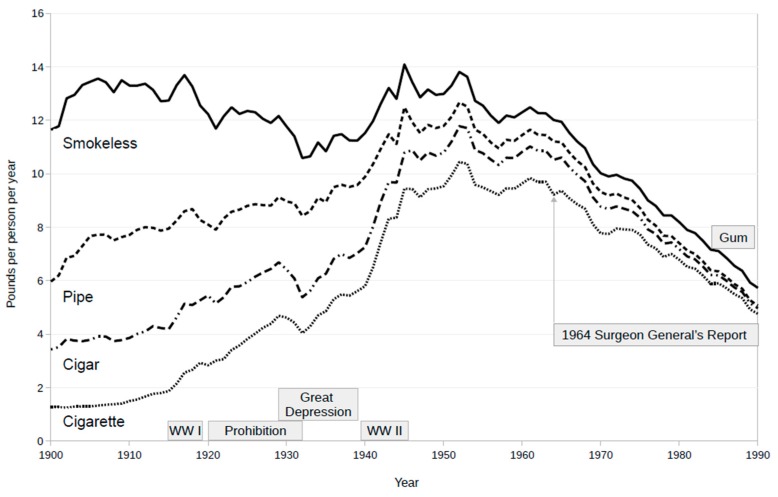
Annual cumulative tobacco product consumption in the USA. Tobacco product sales in pounds per person per year are shown from 1900 to 1990. Derived from Psoter 2001 [4]. The bottom curve represents cigarette tobacco sales. Market share of cigar, pipe and other loose tobacco, and smokeless tobacco products are vertically stacked, so that the solid top line represents total tobacco sales. Nicotine replacement products and electronic cigarettes are not represented. Prescription nicotine gum sales began in 1984 at a rate of $40 million/year, and clearly began to displace tobacco products [5]. WWI is World War I. WWII is World War II. Prohibition was a period when ethanol was illegal to sell for drinking.

First, the nicotine market is far more stable and robust than is apparent from the history of the cigarette market. Cigarette consumption in the United States (USA) rose from a negligible level in 1900 to a post-war peak around 1960, began a precipitous fall after the Surgeon General’s report in 1964 [3], and eventually reached a plateau. It began to decline again after e-cigs appeared. This history of the cigarette market has encouraged a false hope that the prevalence of nicotine use—not just cigarette smoking—can be pushed close to zero. A more complete and much more sobering picture results from summing all tobacco product sales figures [4] over the same time frame, as shown in Figure 1. Tobacco sales hovered between 12 and 14 pounds per person per year from 1900 to 1965, with a dip to 12 pounds during prohibition and below 12 pounds only during the Great Depression. The market merely shifted from chewing, pipe, and cigar tobacco to cigarettes. Per capita tobacco sales dipped into uncharted territory after the Surgeon General’s report: there is no precedent in the USA, or anywhere else, for a collapse in nicotine demand. We believe that a nicotine market is practically inevitable, and we would be pleased to see this market shift toward less risky products [2]. Furthermore, it would be neither surprising nor alarming if increasingly safe nicotine products resulted in modest growth in the number of nicotine users. This would be completely normal in any market. In the nicotine market, growth could result in public health gains to the extent that smoking declines and harm reduction products promote and accelerate this decline.

Second, nicotine use is more complex than commonly appreciated. There are presently four prominent factors to consider when thinking about whether and how individuals use nicotine. These are:

1. *Degree of dependence*, which is heavily influenced by nicotine receptor alleles [6], as well as age of smoking initiation [7]. Conventional smoking cessation aides such as Nicotine Replacement Therapy (NRT) and varenicline target these receptors. Some highly addicted people cannot imagine life without nicotine, even if they use relatively little, but they can use e-cigs instead of smoking.
2. *The rate of nicotine clearance*, which is determined mostly by hepatic cytochrome P450 2A6 (CYP2A6) [8]. CYP2A6 phenotypes range from completely inactive (e.g., persons homozygous for deletion mutations) to hyperactive. The slowest metabolizers probably never smoke more than 7 cigarettes per day (CPD), may respond well to NRT [9], and might even feel intoxicated on high-dose NRT (e.g., 21 mg patches, clinical observation). Normal metabolizers are likely to smoke 10–20 CPD, while very rapid metabolizers smoke more, and may even wake from sleep specifically to smoke (clinical observation). There are no conventional drugs that target CYP2A6, although nicotyrine, found in cigarettes and aging or steeped e-liquids [10], is an irreversible inhibitor of the enzyme and might explain commonly observed changes in dependency measures when smokers switch to e-cigs [11].
3. *Benefits of nicotine use*, which include mood modulation, mental focus, weight control, pain management, and social benefits [12,13,14,15,16,17]. For some of these purposes, chemical alternatives (or complements) to nicotine include caffeine, alcohol, cannabinoids, hallucinogens, benzodiazepines, cocaine, and opiates. Amongst these alternatives, nicotine and caffeine are by far the least damaging to cognition.
4. *Harms of nicotine use*, which depend mostly on the delivery system. Nicotine is associated with minimal health harm for adults at the serum levels that people voluntarily maintain. Children and adolescents are probably vulnerable to central nervous system effects from nicotine (not just smoke) exposure [7], although most of the evidence comes from animal experimental studies or smoking rather than nicotine intake through a cleaner product [18]. Harms of delivery systems are highly variable. Tobacco cigarettes have long been recognized as one of the most dangerous product designs possible. Another nicotine-containing product, snus, is associated with substantially lower harm compared to smoking and this has long been established through clinical epidemiological evidence [19,20]. Although e-cigs are likely to be safer than smoking, there may be some variation in risk between e-liquids and amongst devices. Dual use of cigarettes and e-cigs is common. Although studies have shown similar levels of toxin exposure from dual use as from smoking [21], dual use is often a step toward smoking cessation [22,23], and long-term dual users who substantially decrease cigarette consumption probably reduce their health risks.

The first smokers to quit after the Surgeon General’s report probably included many prosperous, lightly addicted social smokers with slow nicotine metabolism and cardio-pulmonary health concerns. Their experience would not resemble the smoking cessation process for one of today’s stressed and highly addicted fast metabolizers. For current adult smokers, harm reduction is compassionate and practical. Adolescent use would be acceptable only if it was the best way to avoid worse problems.

## 2. The Special Issue

### 2.1. Aerosol Chemistry

User technique is one of the many factors likely to affect e-cig aerosol physics and chemistry. Simulations of e-cig use may require tunable vaping machines. Soulet et al. present a Universal System for Analysis of Vaping (U-SAV), which controls and continuously monitors flow rate and power [24]. It appears to offer excellent control of important parameters, and invites validation studies comparing composition of aerosols produced by the U-SAV against less tunable vaping machines.

Eddingsaas et al. report that two common aerosol collection techniques, silica filtration and cold methanol impingement, collect different compounds from vaporized flavored e-liquids [25]. This result is expected for chemicals that partition to particle phase (low volatility → filter) or gas phase (high volatility → methanol?). Researchers depending on either technique in isolation will miss some aerosol components. Furthermore, a number of compounds were found in aerosols that were not present in the e-liquids, and vice versa. For instance, mango-flavored e-liquid contains hexyl acetate that is not captured by filtration or impingement. The fate of this compound is unknown at present. It may have reacted with another compound on vaporization to form a compound only seen in the aerosol, or it may have been missed by both collection methods. The authors recommend online thermal desorption as a follow-up collection technique. Research in this field should expand in order to identify the most appropriate methods for evaluating different e-cig emissions.

### 2.2. Adolescent Use

Kinnunen et al. found that risk factors for repeated e-cig experimentation amongst adolescents in Helsinki, Finland, were very similar to risk factors for smoking at least 50 cigarettes [26]. Factors putting adolescents at risk for both included having been drunk, occasional or regular energy drink use, less-than-excellent academic performance, and parental smoking. Protective factors included sports and extracurricular activities. Family disruption was a risk factor in univariate but not multivariate models. This suggests either that this particular trauma does not provoke nicotine experimentation, or that it is associated with developing another risk factor that has a stronger correlation with nicotine experimentation. It should be noted that the strongest predictor of e-cig use was smoking conventional cigarettes (both daily and occasional smoking).

Although risk factors for experimenting with e-cigs and cigarettes may be similar, a small telephone survey of high-risk adolescents by Chen et al. agrees that initial experiences are not [27]. In findings consistent with a large printed survey of unselected 6th through 10th grade students [28], cigarettes had more negative effects and were less calming than e-cigs, but delivered a better “buzz”. The studies also agree that adolescents’ positive and negative experiences with cigarettes were associated with increased and decreased odds of smoking, respectively, but initial experiences with e-cigs had no association with subsequent use.

Bauld et al. examined 5 surveys of adolescents in the UK, and find again that “most e-cigarette experimentation does not turn into regular use, and levels of regular use in young people who have never smoked remain very low [29].” As they remark, the gateway hypothesis suggests that smoking rates in young people should increase along with e-cig use, but the observed trend is more consistent with competition between e-cigs and cigarettes. The findings of this study suggest the need to differentiate experimentation from frequent/regular use and measure separately these patterns of use in order to better and more accurately identify the public health impact of e-cigs in the population.

Kinouani et al. conducted a series of four online surveys of 2720 mostly French college students in the i-Share study, including 1305 (48%) smokers, at 6-month intervals [30]. Among 241 former smokers, e-cig past (146) and current (29) use was common. In contrast, e-cig experimentation was common (153) among the 1160 non-smokers, but current use was quite rare (4). Among the few smokers reporting a strong desire to stop smoking (70 of 585), over 80% had tried e-cigs. Although the response rate limits conclusions somewhat, the results show that e-cigs are tried frequently, often out of curiosity but also as a smoking alternative, are often used only transiently as smokers quit, but cannot or do not replace cigarettes for some smokers who want to quit.

### 2.3. Vape Shops

Ward et al. conducted a small qualitative substudy of the ongoing Electronic Cigarette Trajectory (ECTra) study of e-cig users in the East Anglia region of England [31]. The substudy focused on comparing support for smoking abstinence from private vape shops and the Stop Smoking Service (SSS) of the National Health Service. Not surprisingly, e-cig users reported that vape shops were very attentive to their needs related to e-cig use. Nevertheless, it is noteworthy that they perceived the private vape shops to fill a large gap in knowledge and attention from the public SSS. The study should prompt and inform formal trials of vape shop support for smoking cessation.

Yu et al. found that local vape shops were often unaware of pending regulation in the U.S., perhaps because they struggled just to stay open: 20% of the sample closed over the course of a year [32]. The shops also had opportunities to improve employee and customer safety regarding e-liquid spills.

### 2.4. Switching and Smoking Cessation

Truman et al. report serial surveys of adult e-cig users in New Zealand [33], where there are no vape shops, but consumers can legally order e-cigs from other countries. Typical respondents smoked more than 10 CPD and became dual users of less effective e-cig designs. Most eventually reduced or stopped smoking; small numbers of ex-smokers and non-smokers began using e-cigs, but none became smokers. Half of respondents reported that time to first nicotine in the morning increased with e-cig use, whether or not they continued to smoke; most of the remainder reported no change. Once again, this is a change in a dependence measure that cannot be explained by nicotine absorption alone. The regulatory environment in New Zealand appears to be changing, and it would be interesting to see how the patterns of e-cig use change once products will become readily available in the local market.

Adriaens et al. report a survey of adult Dutch e-cig and dual-users, looking for distinguishing features of the two groups [34]. Dual users were slightly but significantly less likely to report that e-cigs curbed their desire for a cigarette, and more likely to report problems with e-cigs. They preferred cigarettes to e-cigs in specific situations, including stressful times, after eating, on awakening, and when drinking alcohol. The dual users reported respiratory health issues much more frequently than e-cig users (presumably a result rather than a cause of dual use, that could be mainly attributed to continuous smoking compared to smoking cessation in the e-cig group). The mean response to the statement, “I feel more addicted to nicotine since vaping,” again suggests that half of respondents felt equally or less addicted to nicotine since starting to use e-cigs, whether or not they continued to smoke.

Rodu and Plurphanswat report on the Population Assessment of Tobacco and Health (PATH) study during 2013–2014 [35]. Their analysis found that smoking cessation attempts using e-cigarettes were less common but more successful than unassisted attempts or attempts relying on social support, NRT, or prescription drugs.

### 2.5. Consumer Issues

Sears et al. found that a disturbing 74% of 567 undergraduate students at a midwestern college thought that the USA Food and Drug Administration designation, “Generally Regarded as Safe” (GRAS), meant that a substance would be safe to inhale [36]. These are not the well-informed consumers needed for an efficient market.

Local vape shop customers are convenient subjects for qualitative research, pilot studies, and to answer some narrow questions. However, this population excludes smokers who tried and rejected e-cigs, former smokers who transiently used e-cigs, and current users who rely on convenience stores, pharmacies, or Internet vendors for their e-cigs and e-liquids. Van Gucht et al. reassuringly found that 203 customers of an online Dutch e-cig vendor at least share previously described characteristics of local vape shop customers [37]. For instance, nearly all were former smokers, many viewed e-cigs as superior to conventional smoking cessation aids, most preferred e-cigs to cigarettes, and 84% felt that their health had improved since they started using e-cigs. Although these Internet customers resemble brick-and-mortar store customers, both populations may be different from the general population of smokers and convenience store customers.

Driving home this point, McKeganey and Dickson queried 650 smokers in contact with the Forest smokers’ rights organization in the UK, and found that 95% said that they smoke because they enjoy smoking: 41% had never tried e-cigs [38]. The 16% who were dual users enjoyed cigarettes more and often used e-cigs “to pass through severe smoker hostile places.” Only 3% of the sample identified e-cigs relative safety as a positive attribute, and many more anticipated that e-cigs would eventually be found to cause significant harm. The authors conclude that, “If e-cigarettes are going to appeal to a much wider range of smokers, it will be necessary for the vaping experience to be at least as enjoyable as smoking (in terms of smokers’ perceptions) and very probably more enjoyable than smoking”. Successful harm reduction will require further product innovation and better public understanding of relative risks.

### 2.6. Policy Issues

Levy et al. analyzed 161,360 responses to the 2014 Tobacco Use Supplement–Current Population Survey in the USA, and correlated state tobacco control policies and individual characteristics with e-cig, smokeless tobacco, and cigarette use [39]. Effective state tobacco control measures were negatively correlated with e-cig use (because e-cig users nearly always start as smokers), while specific regulations limiting or taxing e-cig use have small if any effects on e-cig use.

## 3. Discussion

### 3.1. What We Learned

The observational case for e-cigs as smoking cessation aids is as strong as ever. There is clearly a large group of smokers for whom e-cigs are superior to conventional aids. Nevertheless, there is another large group for whom e-cigs are not interesting or do not work. Current e-cigs will not quickly end smoking, not because they are a gateway to smoking, but because they are not effective enough for every smoker.

Adolescents with smoking risk factors sometimes become e-cig users, but their usual history is that they smoke first. The data are consistent with the hypothesis that adolescents in search of thrills or solace try a variety of risky behaviors, among which e-cigs are one of the least-damaging options. Nevertheless, even this choice could portend a lifelong change in neural function. We need other ways for adolescents to find purpose and contentment.

E-cig and dual users experience about the same declines in some dependence measures that could reflect nicotine clearance. This hints at comparable changes in CYP2A6 activity in e-cig and dual users. Dual users appear to smoke in response to particular cues. If these findings are confirmed, we may need to focus attention on the central nervous system to understand dual use.

At least some vape shops are useful for educating and encouraging new e-cig users. Vape shop personnel can be more knowledgeable than medical staff about both smoking and e-cigs. Online support is also effective.

### 3.2. What We Want to Learn

There is a lot more to know about e-cig aerosols. We know little about particle evolution in vivo, including dynamic partitioning of semi-volatile compounds between particles and gas phase, which will affect deposition patterns, nicotine delivery, and risk. An efficient process for evaluating and monitoring the myriad flavors could be useful. Additionally, the impact of relatively new developments on safety, such as the substitution of silica wick with cotton and the availability of temperature control, have not been studied. Bystander exposure and health effects will be small but deserve evaluation.

Comparative nicotine metabolism in e-cig users and smokers deserves study. The behaviors repeatedly documented in this special issue [27,30,34] and previous work [40,41,42] cannot be explained by nicotine absorption alone, no matter how efficiently an e-cig aerosol delivers nicotine. Furthermore, the efficiency of current e-cig designs does not explain how older e-cigs changed conventional nicotine dependence measures. These changes continue to suggest a major alteration of nicotine pharmacokinetics, such as creation of a nicotine reservoir or impaired clearance, for instance by the inhibition of CYP2A enzymes [10].

The utility of e-cigs as smoking cessation aides remains uncertain, with observational and anecdotal evidence of value, and weak evidence from randomized trials [43]. At least moderate-sized randomized controlled trials are needed to compare e-cigs to conventional smoking cessation options. However, e-cig use is a behavioral intervention, and randomized trials so far have failed to take into account issues such as flavors preferences, different nicotine levels according to personal need, and throat hit sensitivity. Thus, randomized trials should be designed in a more “unconventional” way compared to trials for medications and should also consider the rapid evolution and development of better-performing devices [44]. In addition to tracking smoking status and nicotine use, trial designs ought to collect data on illness; costs to users, employers, and insurers; and quality of life. Trials ideally would plan to determine if cessation aide failures are associated with genetic, clinical, environmental, or intervention variables, or interactions thereof.

At least one efficacy trial is needed in which the e-cig arm offers tank-style e-cigs and realistically managed e-liquids, and includes vetted vape shops and Internet sites for counseling and social support. The conventional arm should deliberately tailor state-of the art NRT and prescription options, marshal friends and families for social support, and offer formal smoking cessation classes and physician visits.

Comparable but distinct effectiveness trials also are needed. At least one of these should employ methods suitable for a busy primary care office, e.g., recommend smokers to e-cigs or a conventional treatment, with passive recommendations for finding advice and support from vetted sources. Such pragmatic trials are expected to provide even more valuable information than randomized controlled trials about the impact of e-cigs on smoking status in daily clinical routine.

There are opportunities to improve observational studies. Most surveys related to nicotine use should include questions that attempt to classify respondents’ nicotinic receptor status and nicotine clearance rates, and attempt to identify and rank the common reasons for using nicotine. The amount of nicotine used is easily calculated from volume and concentration, and should be evaluated as a measure of nicotine use. It will be instructive to know whether or not this correlates with addiction, clearance, and behavior. Additionally, use patterns should definitely consider frequency of use. While “ever use of an e-cig” is of minimal interest per se (it is very unlikely to be associated with any risk and equally unlikely to result in smoking behavior changes), the definition of current use as any past 30-day use has also been shown to include a lot of experimenters [45]. Some harm from e-cig aerosol inhalation is likely, but usually will be overwhelmed by the benefits of smoking cessation. Large data sets with detailed information on changes in smoking and amounts of e-liquid and nicotine consumed may allow health outcome prediction models to disentangle the benefits of smoking reduction and harms of e-cig aerosol inhalation.
